# A global dataset of surface water and groundwater salinity measurements from 1980–2019

**DOI:** 10.1038/s41597-020-0562-z

**Published:** 2020-07-13

**Authors:** Josefin Thorslund, Michelle T. H. van Vliet

**Affiliations:** 1 Department of Physical Geography, Utrecht University, P.O. Box 80115, 3508CB Utrecht, The Netherlands; 2 Department of Physical Geography and the Bolin Centre for Climate Research, Stockholm University, SE- 106 91 Stockholm, Sweden

**Keywords:** Environmental monitoring, Hydrology, Element cycles, Geochemistry, Environmental impact

## Abstract

Salinization of freshwater resources is a growing water quality challenge, which may negatively impact both sectoral water-use and food security, as well as biodiversity and ecosystem services. Although monitoring of salinity is relatively common compared to many other water quality parameters, no compilation and harmonisation of available datasets for both surface and groundwater components have been made yet at the global scale. Here, we present a new global salinity database, compiled from electrical conductivity (EC) monitoring data of both surface water (rivers, lakes/reservoirs) and groundwater locations over the period 1980–2019. The data were assembled from a range of sources, including local to global salinity databases, governmental organizations, river basin management commissions and water development boards. Our resulting database comprises more than 16.3 million measurements from 45,103 surface water locations and 208,550 groundwater locations around the world. This database could provide new opportunities for meta-analyses of salinity levels of water resources, as well as for addressing data and model-driven questions related to historic and future salinization patterns and impacts.

## Abstract (summary)

**Table:** 

Measurement(s)	electrical conductivity
Technology Type(s)	water monitoring station • water quality sampling
Factor Type(s)	date • geographic location
Sample Characteristic - Environment	surface water • groundwater
Sample Characteristic - Location	global

Machine-accessible metadata file describing the reported data: 10.6084/m9.figshare.12455954

## Background & Summary

Freshwater salinization is a growing water quality challenge, affecting both surface and groundwater resources^1,2^. Salinization of freshwater resources may have natural causes, arising from weathering, atmospheric deposition and saltwater intrusion, but rising salinity also occurs due to human activities, such as land alterations, road salts (for de-icing) and irrigation return flows^3^. High salinity levels can negatively impact both sectoral water use, including drinking water supply and irrigation, as well as biodiversity and ecosystem health^4–6^. Although increasing attention is being payed towards problems of freshwater salinization, assessing its extent and magnitude is still challenging and several research gaps remains^7,8^.

Improving water quality is a central part of the UN Sustainable Development Goals (SDGs) and data collection and sharing have been communicated as important steps for reaching associated water quality targets^9,10^. Access to reliable water salinity data is critical for increased understanding of salinity issues and its drivers, and for developing efficient management strategies^11,12^. In addition, using observed salinity data in modelling approaches can contribute to better process understanding and in reducing model prediction uncertainty, enabling better water quality projections under global change^13,14^. Although the number of studies sharing salinity datasets are increasing^15–18^, few assessments extend to the global scale, and even less target both the surface and groundwater systems. In addition, salinity data is often scattered and non-harmonized, both in terms of reported parameters, units, and spatio-temporal resolution. This complicates comparison of information across scales.

To support scientists and others working on freshwater salinity-related topics, we here provide a global, harmonized salinity database, comprising salinity monitoring data of both surface and groundwater components. We collected and combined observational data, focusing mainly on electrical conductivity (EC), which is the most commonly monitored salinity parameter globally. For groundwater, we also included a few additional datasets of total dissolved solids (TDS), which were converted into EC for comparisons across sites. The data was collected from a suite of sources, including local, regional and global water quality databases, governmental organizations, river basin management commissions, water development boards and individual research projects. We included all surface water monitoring stations with at least 30 measurements, and all groundwater stations with measurements and depth information, within the selected time period of 1980–2019.

**Fig. 1:**
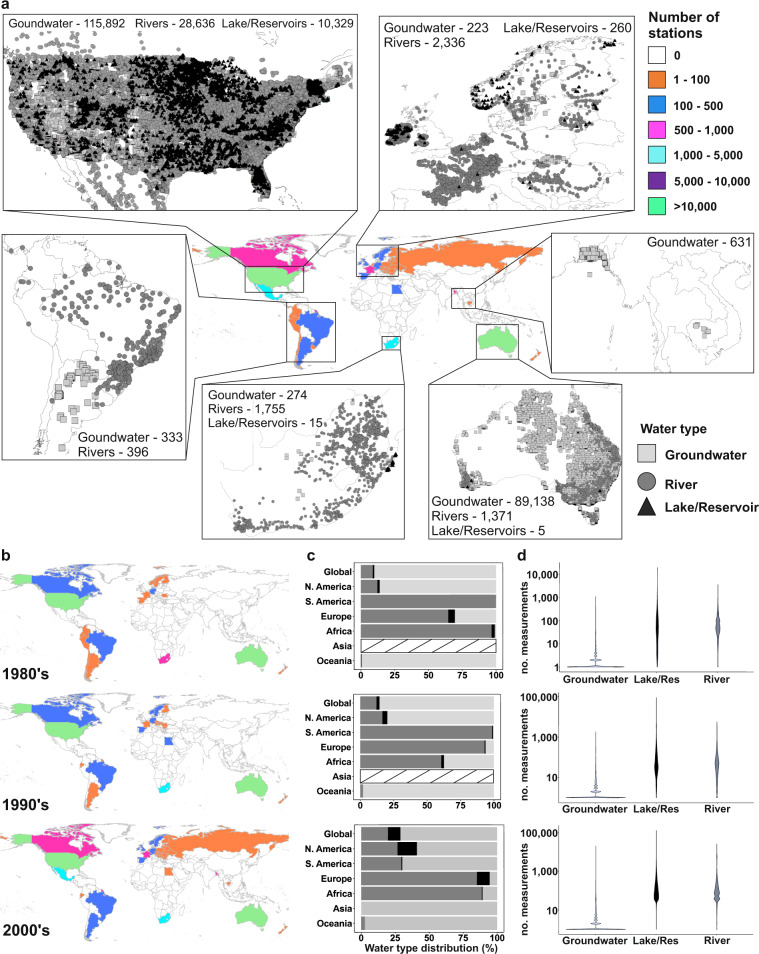
Global overview of station density and measurement distributions. The global map of panel (a) shows the total number of stations per country with electrical conductivity (EC) observations included in our database, over the full data period (1980–2019). The zoomed panels highlight high-density station regions of each continent, whereas the numbers given for each water type is the total number of stations for associated continent. Panel (b) shows number of stations per country for the different decades included in the database (1980–1989, 1990–1999, 2000–2019). Panel (c) shows the distribution of sampled water types (as percentages of total samples) over the three decades, per continent. No data is represented as striped columns. Panel (d) shows violin plots of the distribution of number of measurements, per water type, over the same time periods.

The resulting database contains more than 16.3 million EC measurements, from around 250,000 locations around the world, divided into 34,494 river locations, 10,609 lake or reservoir locations and 208,550 groundwater locations (Fig. 1). Though measurement data was found for all continents, station density and sampling frequency varies greatly, both in space and time. For example, station density is generally highest for North America and Australia (green color of Fig. 1a,b), and overall lowest for Asia and Africa (white color of Fig. 1a,b), with the exception of South Africa that has a high station frequency (turquoise color of Fig. 1a,b). Station density has commonly increased over time, particularly for Europe and parts of South America. The distribution of sampled water types also varies between continents and over time (Fig. 1c). For example, for Asia, no measurements were found for the 1980s and 1990s (striped bar color), and during the 21^st^ century, only groundwater data was reported (light grey bar color). For Europe on the other hand, more groundwater than surface water measurements were obtained in the 1980s than later on. However, for the majority of the sampled water types of this database, the distribution has not changed substantially over time (Fig. 1c). Regarding the number of measurements per water type (Fig. 1d), this is also rather constant throughout time, with groundwaters having an overall much lower sampling frequency than surface waters. Groundwater locations were on average sampled four times, while river, respectively, lake/reservoirs on average contain 321 and 417 samples per station.

This database provides a starting point for global, open-source salinity observational data in surface and groundwater systems and can assist data and model-driven studies at cross-regional to global scales. The database can for example be utilized for assessing (i) spatial and temporal patterns of freshwater salinization, (ii) its impact for ecosystem health and sectoral water use, (iii) estimations of drivers of freshwater salinization across scales, and for (iv) calibration and validation of surface and groundwater salinity models.

## Methods

### Selection criteria

Salinity is the measure of the concentration of dissolved (soluble) salts in water from all sources, and it can be measured by a range of parameters (including dissolved solids fractions, total dissolved solids, chloride, electrical conductivity, salinity) and units (including ppm, mg L^−1^, µS cm^−1^, dS m^−1^). A primary data collection focus here was given to EC measurements, since this is the most widely reported salinity parameter, and a main aim of this database is to provide comparable data across various scales. However, total dissolved solids (TDS) is also a common salinity parameter, particularly for groundwater quality measurements. The relationship of TDS and EC is correlated and can be determined using a conversion factor^19^. Regional conversion factors have been shown to produce better correlations than global factors, since the relationship between EC and TDS depends on a range of factors that may vary spatially, e.g. with climate, temperature, dissolved ion concentrations and ionic strength^20^. Thus, for optimizing data inclusion, a dataset containing TDS measurements was included, but only if a regional conversion factor could be found in the literature (see Methods and Technical Validation for further description on conversion and correlation analyses).

**Fig. 2:**
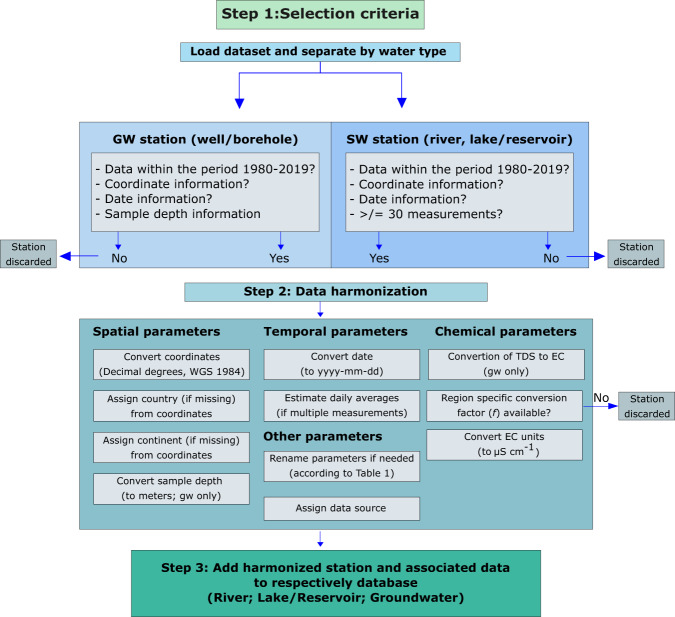
Data selection and harmonisation flowchart. The figure illustrates the processing and harmonizing steps of each dataset (divided into surface and groundwater parts) after initial data collection.

Multiple selection criteria were applied for each monitoring location and water type sampled. Surface waters were divided into the following categories: (i) river; and (ii) lake/reservoir. A sampling location was included if there were at least 30 measurements within the selected time period (1980–2019). For groundwater, we included all measurements at each location, if reported sampling depth information was available. The reason for this less stringent sampling frequency criterion for each groundwater location was due to the general limitation of high frequency groundwater monitoring compared to surface water monitoring^21,22^. Additionally, low temporal resolution groundwater data could provide valuable input for first order salinity assessments, model calibration and/or hypothesis testing^23^. An important variable for interpreting groundwater EC is however sample depth, since this has large implications on, for example, withdrawal depths for different sectoral water use, as well as for estimation of the freshwater/saltwater lens^24^. This thus motivates the depth availability criterion over sampling frequency for groundwaters. In addition to these criteria, all samples also had to have date and coordinate (latitude, longitude) information for qualifying inclusion in the database (see Fig. 2 for a schematic flowchart of the data selection and processing steps).

### Data collection and sources

Data was collected from both surface water and groundwater monitoring locations using a combination of data sources, including: (i) global datasets, (ii) regional datasets, and (iii) individual river basins and groundwater aquifers datasets. The regional data includes datasets spanning multiple river basins and/or groundwater aquifers, both within the same region, but also cross-regionally. Most of these data are provided by governmental organizations or cross-regional data portal platforms under environmental protection agencies or National water quality monitoring programs. The local/individual basins datasets consist of monitoring data for individual basins and were usually found through governmental agencies, river basin management commissions, research organizations, as well as provided by individual researchers. Each data source is listed and described shortly below (the data source abbreviations were defined by us, for easy reference to the database terminology). A full list of the corresponding data (including their spatial and temporal resolution) for each of these sources (including their URL), divided by water type, is given in online-only Table 1.

For the here presented database, we focused on combining and harmonizing EC datasets from already available, open data sources. The reason for this is that EC is often included in broader environmental monitoring websites and/or water quality datasets, which are not identifiable as salinity datasets, but rather in general water quality terms. We thus wanted to extract the salinity data component, and facilitate the reuse of harmonized EC data for salinity-specific applications. Most of the dataset included in our database have original licenses that permit unrestricted reuse. Where this was not the case, or if information was lacking, we requested and were granted permission from the data owners to release the data under the CC-BY license.

Although we acknowledge the potential of valuable datasets in the scientific literature, this was not a data focus type, since this requires a different data search and extraction approach. We only incorporated pre-extracted datasets from literature reviews and synthesis when shared from individual researchers (reached through communication within our research community, e.g. during workshops and conferences and within own networks and communication channels). The following subsections provide an overview of the global, regional and local salinity datasets included in our developed database.

**Global salinity dataset**

The Global River Chemistry Dataset (***GLORICH***) includes multiple water quality parameters for river locations around the world, assembled by researchers from Hamburg University^25,26^. This data is publicly available and was downloaded as a zip file from PANGEA. The dataset includes 1.27 million samples of major compounds, nutrients, carbon species and physical properties. We extracted Specific Conductivity data (another terminology for EC) from the “hydrochemistry” csv file and paired it with station information (“Sampling_locations” file), for all stations that fulfilled our selection criteria.

**Regional salinity datasets:**

1. Data for Europe was collected from the European Environment Agency’s water quality database; ***Waterbase****.* Waterbase contains multiple water quality parameters for rivers, lakes and groundwater bodies throughout Europe. We extracted relevant EC and station information data using the raw disaggregated water quality data file: “Waterbase_v2018_1_T_WISE4_DisaggregatedData” and the parameter code for EC (“EEA_3142-01-6”, specified as *Specific Conductance*). The water types were identified and distinguished from the column *parameterWaterBodyCategory*, where “RW” is river, “LW” is lake and “GW” is groundwater location. Site information was extracted from the file: “Waterbase_v2018_1_WISE4_MonitoringSite_DerivedData”. The groundwater EC data was matched with depth information, using the *parameterSampleDepth* parameter.
2. The Water Quality Portal (***WQP***) for surface and groundwaters across the United States contains a range of water quality data for surface and groundwaters across the US. The data portal is established by the United States Geological Survey (USGS), the Environmental Protection Agency (EPA), and the National Water Quality Monitoring Council (NWQMC). The data originated from state, federal, tribal, and local agencies. Data was downloaded in bulk, for Specific conductance, for all available sites included under the search criteria (i) *streams*, (ii) *lake, reservoir, impoundment* and (iii) *subsurface*. Station information was additionally downloaded and paired with the salinity data.
3. Groundwater data for the US was also gathered from the Dissolved-Solids Dataset (***Qi & Harris 2017***)^27^, by downloading the “Dissolved solids” csv file and combining it with depth information from the “AquiferDepthSources” excel file. This data is published by the ScienceBase Catalog, provided by the USGS and contains EC (and other geochemical) data that was collected with the purpose of assessing brackish groundwaters across the United States. The original dataset contains a compilation of water-quality samples from 33 sources for almost 384,000 groundwater wells across the continental U.S., Alaska, Hawaii, Puerto Rico, the U.S. Virgin Islands, Guam, and American Samoa, dating back to the early 18^th^ century.
4. Groundwater data from Colorado was collected from the Department of Agriculture and Agricultural Chemicals & Groundwater Protection section (***Co Gov***). Data was downloaded directly from the site using a search query of *statewide inorganic quality monitoring data*, and selecting the parameter *Specific Conductance (Lab)*, for all available years. Site coordinate (latitude, longitude) information was not available online, but when requested via email, it was submitted to us, by their groundwater monitoring specialists (Karl Mauch, personal email communication). In addition, data on well sampling depth estimations were also provided via email, and the *perforated interval* measure (the interval between top and bottom of perforated section where the pump is installed) was recommended and used as depth information.
5. Groundwater data from California was downloaded from the GeoTracker Groundwater Ambient Monitoring and Assessment Program (***GAMA***), provided by the California state open data portal. The dataset includes multiple groundwater quality data from the GAMA Domestic Well (DW) and Priority Basin (PB) programs, covering locations throughout the state. The column “well_depth” was the only depth information available, and was included (and converted from feet to meters) as the *Depth* parameter.
6. Groundwater monitoring data from the Ohio Environmental Protection Agency (***Ohio EPA***) was downloaded from their ambient groundwater monitoring program. Monitoring of groundwater wells was established in the late 1960s and today covers more than 300 wells. Also here, the “well_depth” parameter was the only depth information available, and was included (and converted from feet to meters) as the *Depth* parameter.
7. The groundwater database from the Texas Water Development Board (***TWDB***) was also utilized to download water quality data. EC data was downloaded in bulk by groundwater aquifer (in total nine datasets). Well depths were converted from feet to meters and where multiple measurements for the same day and well was reported, daily averages were calculated. A total of 404 wells fulfilled the selection criteria and were included in the main groundwater database.
8. Data for South Africa was collected from the Department of Water and Sanitation (***DWS***), Republic of South Africa^28^. Both surface- and groundwaters are monitored, as a part of their National Chemical Monitoring Program. Monitoring stations and their data can be viewed and downloaded through the Water quality data exploration tool. However, due to the large amount of data for surface waters, we requested and recieved raw water quality data from the Resource Quality Information Services national monitoring programs for specific rivers and dams, through E-mail.
9. Surface water monitoring data for a large part of Australia is provided by the Australian Government, Bureau of Meteorology (***AU Gov***). Data can be queried at the Water Data Online portal, and search criteria can be specified. Conducted search criteria of all stations with EC data resulted in 1,333 stations. However, since data can only be downloaded as one by one station, we sent an email through the help desk system requesting a bulk download of all available data. The data was then provided as daily means recorded at midnight and as csv files (one file per station), with a metadata summary file included (with station information). From this, all files were combined and stations that fulfilled the selection criteria were then included in the main database. The separation between river and lake/reservoir locations were determined from the datafile “long_name” column, which always included the water type as well as the actual name of the monitoring location.
10. Surface water data for Australia was also synthesized from the Queensland Government Open Data Portal (***QLD AU Gov***). Data from ***QLD AU Gov*** was collected from the ambient estuary water quality monitoring program, which includes tidal rivers, streams and inshore waters of Central Queensland, monitored from 1993–2013. Data is available for 12 different drainage basins, reported as *Specific Conductance at 25 °C*. Data was downloaded as individual csv-files for each drainage basin (containing multiple sampling locations), and then combined and extracted according to the selection criteria.
11. Groundwater data for Australia was gathered from the Australian Government Bioregional Assessment Program (***BAP***). The data is provided through a collaboration between the Department of the Environment and Energy, the Bureau of Meteorology, CSIRO and Geoscience Australia. The dataset contains EC measurements of groundwater bores in the Namoi sub-region. The data is collected from groundwater bores that fell within the data management acquisition area as provided by the Bioregional Assessment to the Namoi NSW Office of Water. All data were downloaded in one csv-file.
12. Another groundwater dataset from Australia was collected, using the groundwater data portal from ***WaterConnect****,* which provides data from the Department for Environment and Water, for South Australia. Data was here queried by region, and then one file containing EC data for all sampled wells and one file containing site information were downloaded, for each region (in total 12 regions). The “Latest_Depth (m)” was used for depth information and all stations with both depth and EC measurements for a given data were included.
13. Additional groundwater data from Australia was downloaded using the Australian Groundwater Explorer tool (***AU GwEX***). Data was here search for by parameters *Water level* and *Salinity* and downloaded by region (in total 8 regions) and combined. Water levels and EC data was linked to the NGIS bore data to get the location and attributes of the measurement wells.
14. Data for New Zealand was gathered from New Zealand’s Hydro Web Portal for Hydrometric and Water Quality data (***NIWA***). This platform provides river water quality data under the National Institute of Water and Atmospheric Research. Data was queried by searching for all available data under the parameter *conductivity* and *time-series*, in their map interphase (resulting in 77 locations of timeseries data). Each dataset was then added for bulk export, using the export tab and a download link, via the map-interface platform.
15. Surface water quality data from the Government of Canada (***Ca Gov***) was downloaded from the *National Long-term Water Quality Monitoring Data* portal. The data include both rivers and lakes monitored for a set of physio-chemical variables, including specific conductance. Data was downloaded as csv-files.
16. River data was also synthesized from the Government of Ontario for multiple rivers, monitored between 2000–2016. The data is collected by the Provincial (Stream) Water Quality Monitoring Network (PWQMN), who measures water quality in rivers and streams across Ontario. Data was downloaded as individual excel files for each year, and then combined with site information.
17. Groundwater data from Argentina was downloaded from the repository of open public data of the Argentinian Republic (***Dat.ar***). The data is provided by the Federal Groundwater Information System SIFAS-SISAG and contains groundwater well measurements from April 2015. The data was downloaded as a main csv-file and translated from Spanish.
18. Groundwater data was also collected from Cambodia, using the online well database of Cambodia (*WellMap*). *WellMap* is an initiative of the Ministry of Rural Development of Cambodia, supported by the Water and Sanitation Program of the World Bank (***WSP***). The database is provided as a Microsoft Access Database and consists of water quality data collected from rural wells throughout the Country. Data was queried and extracted using the *RODBC* R package, that allows R interfacing to database systems. UTM coordinates were re-projected and converted to latitude and longitude, as decimal degrees, using the functions “proj4string” and “spTransform” in R.
19. Data from Mexico Government (***MX Gov***), was downloaded and translated (from Spanish) from one main csv-file, containing both water quality and site information data. The data included both surface water locations (original classification was *rivers*, *streams, dams*, which were reclassified to the here used terminology) and groundwater locations, monitored since 2012.
20. Groundwater data from Bangladesh was provided by M.M. Rahman (TH Cologne, University of Applied Sciences, Institute for Technology and Resources Management in the Tropics and Subtropics). The data was collected and shared by M.M. Rahman, and include electrical conductivity and depth data synthesized from both literature and governmental sources (see specifications and references in online-only Table 1).
21. Groundwater EC and level data from the Swedish geological Survey (SGU) was downloaded, on a county basis, for all 21 counties in Sweden, from environmental monitoring data. EC data was extracted from environmental monitoring files, with one file per county (queried using county specific codes and a URL link to each dataset) and combined with well water level data (downloaded in the same way as the salinity data) using matching coordinates. All stations with water level information were translated to English and were included in the main groundwater database.

**Salinity datasets from individual river basins and groundwater aquifers:**

1. Data for river locations within the Danoube river basin was collected from the Danube River Basin Water Quality Database. This database is provided by the International Commission for Protection of the Danube River (ICPDR) Information System Danubis (***ICPDR***). The database provides geochemical data for the major rivers in the Danube River Basin and waters are sampled at a minimum frequency of 12 times per year. The data was accessed through creating an account, and then performing a data search, for all available years and stations for the conductivity parameter, and exporting the resulting data as a csv file.
2. Data for the lower Murray Darling river basin was accessed through the Water Connect data portal (***Waterconnect***). All stations within the river basin that fulfilled the data selection criteria (six stations) were included and downloaded, one by one (using a combination of the *historical EC daily readings* and the *Site summary* files).
3. Groundwater TDS data for the Nile Delta aquifer (***van Engelen et al****.*)^29^ was provided by Joeri van Engelen. These data include three datasets consisting of TDS measurements, synthesized from literature, collected with the selection criteria of including measurement data from less than 250 m depth. Two of these datasets had unspecific dates, and samples were thus assumed to be from the 1^st^ of each reported month (see further specification of the data in van Engelen *et al*.^29^). The TDS data was then converted to EC, using a regional specific conversion factor, from literature sources (see section *Conversions of TDS to EC* for specifics on how this was done).

### Data processing and harmonization

The overall objective with this database is to facilitate data reuse and research efforts within different fields of salinity research. For this purpose, the harmonization of data was a main part of the database construction. The flowchart (Fig. 2) illustrates the data selection criteria, data processing and harmonization of each sampling location and its associated dataset before it was added to the main database. All processing was done in R, version 3.6.0, using mainly the *data.table* and *dplyr* R packages. First, harmonization and fixing of data with regards to missing values and other uninterpretable field values and/or symbols preventing the appropriate reading of data files (i.e., special symbols like “***” or erroneous changes in field separators, e.g. from “,” to “;”) were done, e.g. by setting it to the standard missing data value (i.e., NA values) and by fixing or excluding rows which could not be read properly. Additionally, assumed erroneous data values for reported salinity values and depth (such as negative values, 999 and 9999, as well as depth values of zero) were removed.

Since information on sampling water type and parameter nomenclature and reported units differs between regions and organizations, we re-classified water types into the three mentioned categories (river, lake/reservoir, groundwater). Where needed, we also re-named and converted other parameters and their associated units, according to the database variables listed in Table 1.

**Table 1:** Variable names and descriptions, including reported units, of the salinity database.

Variable Name	Description	Unit
Station_ID	unique sampling point ID	—
Date	Date of sample	yyyy-mm-dd
Start_date	Date of first sample in record	yyyy-mm-dd
End_date	Date of last sample in record	yyyy-mm-dd
Lat	Latitudinal coordinate of sample location	Decimal Degrees
Lon	Longitudinal coordinate of sample location	Decimal Degrees
Country	Geographic location	—
Continent	Geographic location	—
Water_type	water resource type sampled	(i) Groundwater, (ii) River, (iii) Lake/Reservoir
EC	Electrical conductivity value	µS cm^−1^
TDS	Total dissolved solids value (only groundwater)	mg L^−1^
EC_conv	Converted EC value from TDS and conversion factor	µS cm^−1^
Depth	Depth of groundwater sample	meters (m)
Source	Data source of the dataset. Source links are included in online-only Table 1	—
Coastal_location	Identification if station location is coastal (<10 km from the coastline)	Yes/No
n	Total number of samples for each sampling point	—
median	EC sample median by sampling point	µS cm^−1^
mean	EC sample mean by sampling point	µS cm^−1^
max	EC sample max by sampling point	µS cm^−1^
min	EC sample min by sampling point	µS cm^−1^
sd	EC sample standard deviation by sampling point	µS cm^−1^
median_TDS^*^	TDS sample median by sampling point	mg L^−1^
mean_TDS^*^	TDS sample mean by sampling point	mg L^−1^
max_TDS^*^	TDS sample max by sampling point	mg L^−1^
min_TDS^*^	TDS sample min by sampling point	mg L^−1^
sd_TDS^*^	TDS sample standard deviation by sampling point	mg L^−1^
median_EC_conv^*^	Converted EC sample median by sampling point	mg L^−1^
mean_EC_conv^*^	Converted EC sample mean by sampling point	mg L^−1^
max_EC_conv^*^	Converted EC sample max by sampling point	mg L^−1^
min_EC_conv^*^	Converted EC sample min by sampling point	mg L^−1^
sd_EC_conv^*^	Converted EC sample standard deviation by sampling point	mg L^−1^

Names with ^*^indicate variables which were only included for groundwater samples.

Different spatial and temporal conversions were also made (see Fig. 2). For instance, where multiple measurements per day were available, these were averaged into daily values, using the *data.table* package, and grouping by *Station_ID* and *Date* (see Table 1 for parameter definitions). Depth conversions were also common and included conversions from feet or centimeter to meters. Regarding spatial harmonization, each sample coordinates were converted to decimal degrees and re-projected to WGS 1984, if needed, using the “SpatialPoints”, “proj4string“ and the “spTransform” function of the *rgdal* R-package. If country information was missing, this was assigned from coordinates of each station using the package *map.where*, or extracted from country codes (if available) using the function “countrycode”. Continent information was then assigned from country names, also using the “countrycode” function, by matching country name with continent.

For assisting studies that might be interested specifically in coastal regions and applications, we also quantified if a sampling location was coastal or not. This analysis was done in ArcMap, using the “Near Table” analysis tool. The distance from all sampling locations to the coastline was computed, (using vector data from Natural Earth: https://www.naturalearthdata.com/downloads/10m-physical-vectors/). All locations within 10 km from the coastline were classified as being coastal. The identification of coastal stations was then included in each database summary file, under the column “Coastal_location” (see Table 1).

### Conversions of TDS to EC

We considered the inclusion of additional groundwater data, where TDS measurements could be converted to EC. The relationship between EC and other measured salinity parameters (e.g. TDS) is depending on a range of conditions, such as temperature, climate and concentrations of ionic and undissociated species^18^. This relationship is commonly estimated according to Eq. (1).

1 $$EC=\frac{TDS}{f}$$

where *EC* is in µS cm^−1^, *TDS* in mg L^−1^ and *f* is a conversion factor^19,30^. Commonly, predefined conversion factors without proper site-specific validation are used, but such estimation may be highly uncertain, due to the conditions mentioned above^20^. Instead, it has been shown that the use of region-specific conversion factors may be more representative, since these have been developed from measured relationships between EC and TDS under more local-reginal conditions^19,20^.

Due to reported improved predictability of EC-TDS relationships when using region-specific conversion factors (*f*), we included additional groundwater TDS measurements only for regions with available reported region-specific *f* values. This resulted in the inclusion of three additional groundwater datasets to the final database; one from Idaho^31^, one from California^32^ and one from Egypt^29^. Together these datasets added 3,477 sampling locations and a total of 9,654 measurements to the groundwater database. Both the original TDS data, as well as the converted EC values are included in the database.

For the two TDS groundwater datasets from the United States, TDS was converted to EC using the region-specific conversion factor *f* of 0.65. This conversion factor has been developed for the continental United States, by the US Geological Survey and is widely used cross-regionally within the US^20,33^. For the TDS groundwater data from Egypt (from the Nile delta)^29^, we converted TDS to EC using the region-specific conversion factor *f* of 0.64. This factor value has been derived from local measurement data in the Nile delta itself^34^.

**Fig. 3:**
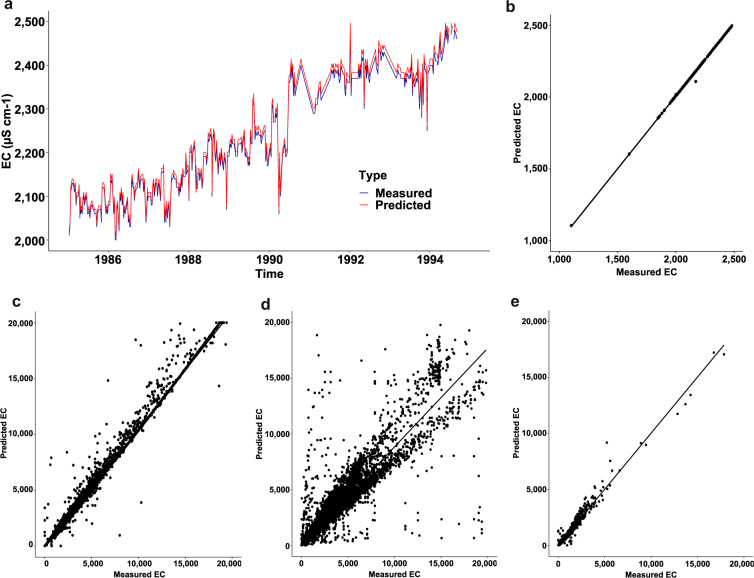
Validation of converted TDS to EC for groundwaters. Time-series plot and scatter correlations of measured vs. predicted electrical conductivity (EC), using regional conversion factors. Panel (a) shows an example time-series from the groundwater station with the highest number of measurements (estimated from the “max” function in R) in Australia (data source: Water connect, n = 538) and panel (b) shows its corresponding scatter correlation (R^2^ = 0.99). Panel (c) shows the correlation between measured and converted EC for the full dataset of all groundwater stations from Water connect (n= 37,819, R^2^ = 0.98). Panel (d) and (e) shows correlations between measured and predicted EC data, for groundwaters in Texas (data source: TWDB, n = 59,985, R^2^ = 0.91) respectively California (data source: GAMA, n = 4,706, R^2^ = 0.98). All scatterplots were done in R, using the “ggscatter” function from the ggpubr package and estimating correlation coefficients using the “pearson” function.

For validation of our approach of predicting EC from TDS, we used regional-conversion factor *f* values on other groundwater datasets that had both TDS and EC measurements reported. These datasets, including data from both the US and from Australia, showed strong correlations between predicted and measured EC (Fig. 3; R^2^ of 0.91–0.99), supporting the approach of using TDS and region-specific conversion factors to estimate EC (see *Technical validation* section).

## Data Records

The salinity database can be downloaded from PANGAEA^35^ and consists of the following 3 categories and associated listed files:

**Category 1**: ***River Data***. This folder contains the full river database, which consists of a csv file with all EC and site related data for each river location. This folder also contains a data summary file, which provides basic EC statistics (median, mean, max, min, sd), sampling summary information (start and end period of measurements, number of measurements) and other station and data information (coordinates, country, continent, data source) for each sampled location (Station_ID).

- *Rivers_ database.csv*
- *Rivers_summary.csv*

**Category 2: Lake/Reservoir Data**. This folder contains the full database for lakes and/or reservoirs EC data, as well as the summary file, in accordance with the descriptions above.

- *Lakes_Reservoirs_database.csv*
- *Lakes_Reservoirs_summary.csv*

**Category 3: Groundwater Data**. This folder contains all groundwater data, and its associated summary file. For the groundwater files, both measured EC, TDS and converted EC are included as separate columns in both the database file and associated summary file.

- *Groundwaters_database.csv*
- *Groundwaters_summary.csv*

For all files, the data source for each station is included, and its associated data link is given in online-only Table 1 and the definitions and units used for each column variable names are given in Table 1. Sample R code, including instructions for reading the database files and for reproducing of figures of this paper, is also available as part of this data record.

## Technical Validation

The converted groundwater EC measurements from TDS are a main source of uncertainty in our database. Thus, to assess the validity of Eq. (1) to predict EC from TDS, we applied the approach on datasets in our database where we could find simultaneous EC and TDS measurements, as well as a corresponding region-specific conversion factor. The validation datasets include one dataset from Australia (from the data source: ***Waterconnect***) and two datasets from the US (from the data sources: **TWDB** and ***GAMA***). For the Australian dataset, we applied the conversion factor, *f* of 0.55. This factor is reported at the Department of Environment and Water, from the Government of Australia and is for instance used for the Murray-Darling basin (AU Gov 2015). As mentioned above, we used the conversion factor, *f* of 0.65 for the US data.

Figure 3 shows different examples of measured versus predicted EC and their correlation, for these groundwater datasets that had simultaneous EC and TDS measurements and a reported region-specific conversion factor. Specifically, figure 3a shows a time-series example of the relation between measured and predicted EC from the Australia dataset, for the station with the highest number of measurements (Station ID: 72559, n = 538). The Pearson correlation scatterplot of measured and predicted EC for this station using the region-specific factor of 0.55 showed a strong positive statically significant correlation (Fig. 3b, R^2^ = 0.99). This strong correlation pattern was also consistent when including all groundwater stations and their associated data from this dataset (R^2^ = 0.98, n = 37,819, Fig. 3c). For the remaining two datasets, one dataset originates from Texas (n = 59,985; Fig. 3d) and one from California (n = 4,706; Fig. 3e). The California dataset show strong positive statistically significant correlations between measured and predicted EC (R^2^ = 0.98). In comparison, the groundwater dataset from Texas is much larger and represent a more heterogenous system than the other locations. This dataset spans larger measurement depths and potentially also larger temperature ranges (no data on this), which may require different conversion factors to improve the results. Given the very large sample size, such effects could explain observed larger bias (both under and over-predictions) in this system compared to the other locations. However, the vast majority of the datapoints are close to the 1:1 line and show strong positive statistically significant correlations (R^2^ = 0.91). Overall, these examples highlight the potential of robust predictability of EC from TDS for groundwater measurements used in combination with regional established conversion factors.

## Data Availability

The data for this study was mainly processed in R (version 3.6.0), but with cross-checking and corrections of spatial coordinates conducted using ArcGIS. Sample R codes, including instructions for reading the database files and reproducing summary files and figures of this paper, is available as part of the data record^35^.

## Online-only Table

**Online-only Table 1:** List of all electrical conductivity datasets that make up the final database, including the number of monitoring stations and total number of measurements, by water type and data source.

Source	Water Type	Nr of Stations	Nr of measurements	Data Reference/URL
AU Gov	River	1,316	4,989,322	http://www.bom.gov.au/waterdata/
AU Gov	Lake/Reservoir	5	16,234	http://www.bom.gov.au/waterdata/
AU GwEX	Groundwater	88,513	746,189	http://www.bom.gov.au/water/groundwater/explorer/map.shtml
BAP	Groundwater	641	641	https://data.gov.au/data/dataset/b5dd0f26-1afa-4a86-8c41-0ed56f86843e
BWDB	Groundwater	511	661	Bangladesh Water Development Board (shared by M.M. Rahman)
CA Gov	River	56	20,252	http://data.ec.gc.ca/data/substances/monitor/national-long-term-water-quality-monitoring-data/?lang=en
Co Gov	Groundwater	217	12,994	https://www.colorado.gov/agconservation/groundwaterprotection
DWS	Groundwater	23	668	http://www.dwa.gov.za/iwqs/wms/data/000key.asp
DWS	River	1,755	485,990	http://www.dwa.gov.za/iwqs/wms/data/000key.asp
DWS	Lake/Reservoir	13	3,359	http://www.dwa.gov.za/iwqs/wms/data/000key.asp
Dat.ar	Groundwater	333	456	http://datar.noip.me/dataset/pozos-sifas
GAMA	Groundwater	2,471	5,837	https://data.ca.gov/dataset/ground-water-water-quality-results
GLORICH	River	2,656	417,196	https://doi.org/10.1594/PANGAEA.902360
GLORICH	Lake/Reservoir	4	1,097	https://doi.org/10.1594/PANGAEA.902360
Hundt *et al*. 2018^*^	Groundwater	3,016	9,182	https://doi.org/10.5066/F72V2FBG
ICPDR	River	117	36,215	https://www.icpdr.org/wq-db/wq/search
MX Gov	River	1,412	53,750	https://www.gob.mx/conagua/articulos/calidad-del-agua
Metzger *et al*. 2018^*^	Groundwater	210	221	https://doi.org/10.5066/F7RN373C
NIWA	River	33	18,708	https://hydrowebportal.niwa.co.nz/Data/
Naus *et al*. 2019	Groundwater	112	112	https://doi.org/10.5194/hess-23-1431-2019
Ohio EPA	Groundwater	348	4,655	https://epa.ohio.gov/
Ontario Gov	River	259	34,567	https://data.ontario.ca/dataset/provincial-stream-water-quality-monitoring-network
QLD AU Gov	River	16	116,880	https://www.data.qld.gov.au/
Qi & Harris 2017	Groundwater	101,235	101,235	https://doi.org/10.5066/F72F7KK1
SGU	Groundwater	133	4,873	https://www.sgu.se/
TWDB	Groundwater	10,963	25,075	http://www.twdb.texas.gov/groundwater/data/gwdbrpt.asp
WQP	Groundwater	658	4,039	https://www.waterqualitydata.us/
WQP	River	25,360	4,708,764	https://www.waterqualitydata.us/
WQP	Lake/Reservoir	10,329	4,389,080	https://www.waterqualitydata.us/
WSP	Groundwater	8	976	https://cambodiawellmap.com/worldbank/maps
Waterbase	River	1,508	79,242	https://www.eea.europa.eu/data-and-maps/data/waterbase-water-quality-2
Waterbase	Lake/Reservoir	260	11,213	https://www.eea.europa.eu/data-and-maps/data/waterbase-water-quality-2
Waterbase	Groundwater	90	6,387	https://www.eea.europa.eu/data-and-maps/data/waterbase-water-quality-2
Waterconnect	Groundwater	1,221	26,704	https://www.waterconnect.sa.gov.au
Waterconnect	River	6	47,462	https://www.waterconnect.sa.gov.au
van Engelen *et al*. 2019^*^	Groundwater	251	251	https://doi.org/10.5194/hess-23-5175-2019

Included is also data references/URL. Data sources marked with a ^*^ indicate TDS datasets, which were converted to EC. All datasets either have original licenses that permit unrestricted reuse, or were granted from the data owners for release under the CC-BY license.
